# Supplementary material for: Inverse Design of Unitary Transmission Matrices in Silicon Photonic Coupled Waveguide Arrays Using a Neural Adjoint Model
Source: ACS Photonics. 2025 Feb 12;12(3):1480–93. doi: 10.1021/acsphotonics.4c02081 (PMC11926960; doi:10.1021/acsphotonics.4c02081)
Supplement: Supplementary file 1 — ph4c02081_si_001.pdf [file ph4c02081_si_001.pdf]

Supporting Information to Inverse Design of Unitary Transmission Matrices in Silicon Photonic Coupled Waveguide Arrays using a Neural Adjoint Model

Thomas W. Radford,*,† Peter R. Wiecha,‡ Alberto Politi,† Ioannis Zeimpekis,¶,§ and Otto L. Muskens*,†

†*School of Physics and Astronomy, University of Southampton, Southampton, SO17 1BJ, United Kingdom*

‡*LAAS, Université de Toulouse, CNRS, 31031, Toulouse, France*

¶*School of Electronics and Computer Science, University of Southampton, Southampton, SO17 1BJ, United Kingdom*

§*Optoelectronics Research Centre, University of Southampton, Southampton, SO17 1BJ, United Kingdom*

E-mail: T.Radford@soton.ac.uk; O.Muskens@soton.ac.uk

Phone: +44 (0)23 80593911

Supporting Information

Network schematic

Figure S1 and Figure S2 show a detailed schematic of the constituent parts of the forward predictor network (S1) and WGAN (S2) which is used after the re-parameterization of training data carried out to allow a gradient optimization on latent vectors from the device geometry space instead of directly on pixel pattern geometries. Residual blocks are integrated into the forward network, which split the output of some layers between both the nearest forward layer as well as a seperate deeper layer, bypassing the intermediate connections.

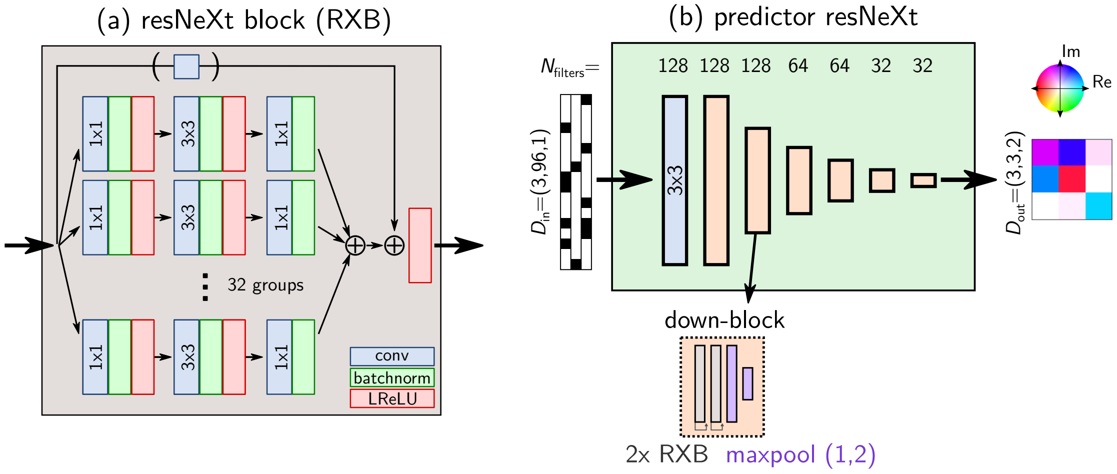

Figure S1: Detailed sketch of the forward network architecture. The transmission predictor is composed of ResNeXt blocks, using grouped convolutions (a). We use a cardinality of 32 and a bottleneck width of 4. For similar computational cost and network complexity, they are known to be more efficient than classic residual blocks.[1] The forward model (b) is trained for 100 epochs using a simple plateau learning rate reduction with a factor of 0.5. Every 25 epochs we furthermore increase the batchsize by a factor of 2, from a starting value of 16, following the suggestion of.[2]

Phase change scattering losses

Sb_2Se_3 posses low losses across both amorphous and crystalline phases when operating at telecommunication wavelengths. The leading loss mechanism in patterned devices is therefore scattering from pixel boundaries and insertion losses to the PCM region. Figure S3

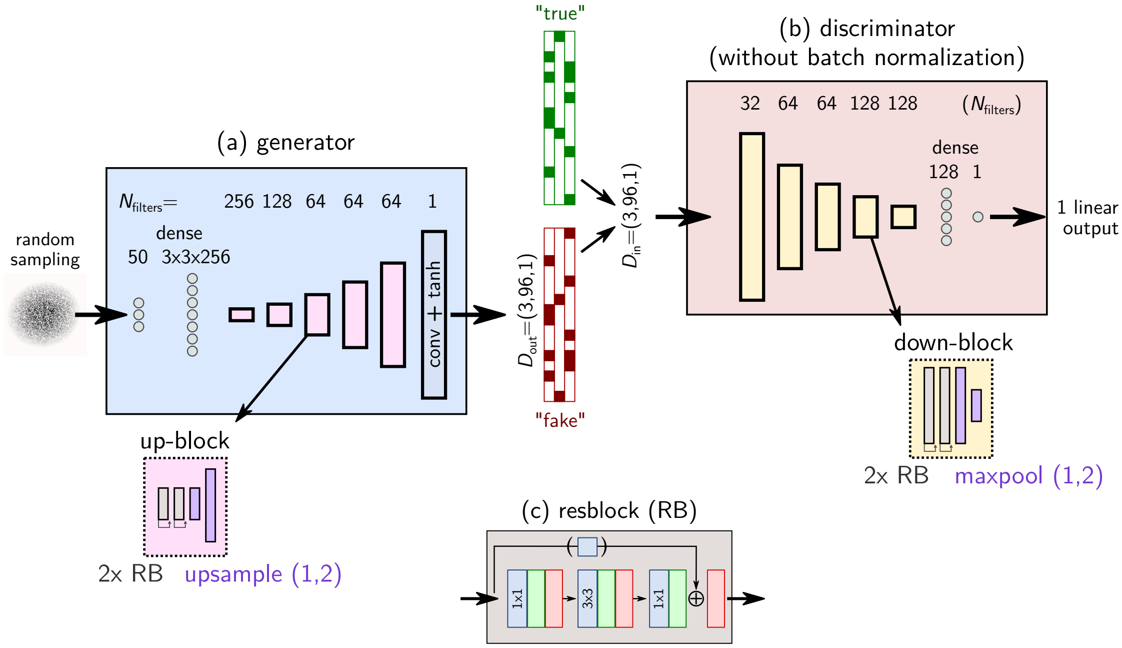

Figure S2: Detailed sketch of the WGAN-GP generator (a) and discriminator (b) architecture, following closely the layout of the original implementation.[3] In particular, we use conventional resnet blocks (c) and no batch normalization in the discriminator. The latter is trained for 3 steps for a single training step of the generator. We train during 15 epochs first with a batch sizes of 64, followed by 15 additional epochs with a batchsize of 128. Both networks are trained using the Adam optimizer[4] with a fixed learning rate of 0.00005.

demonstrates this, pixels are randomly selected and switched increasing light scattered from the device. As $>$ 50% of pixels are switched, the number of amorphous/crystalline boundaries falls and transmission increases again. The maximum recorded drop in device transmission is 8% from its initial input.

Coupled waveguide cross section

Figure S4 Shows a cross section from the coupling region of our simulated device, corresponding to a standard silicon on insulator fabrication process. A silicon wafer is topped with a SiO_2 buried oxide layer, on top of this devices are etched into a further silicon layer. Rib waveguides, spaced by 250 nm, are etched 120 nm into a 200 nm substrate. In the PCM programming region, a 30 nm layer of Sb_2Se_3 is deposited before the entire device is capped with a SiO_2 layer. For presented simulation results the background index of our simulation

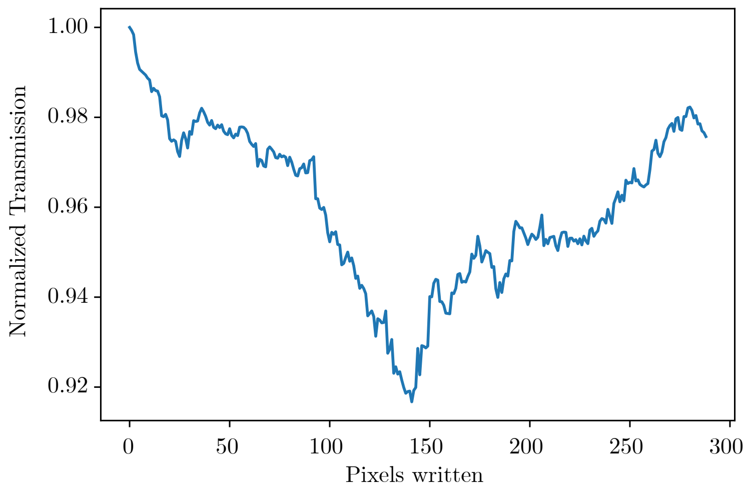

Figure S3: Total device transmission normalised to the unperturbed throughput as a function of number of pixels switched. Switching pixels introduces reflections and scattering losses at the pixel boundaries, decreasing transmission. As $> 50\%$ of pixels are switched the number of these boundaries decreases and transmission increases. The maximum recorded drop in transmission is 8%.

region was set to 1.44, corresponding to that of SiO_2 at 1550nm.

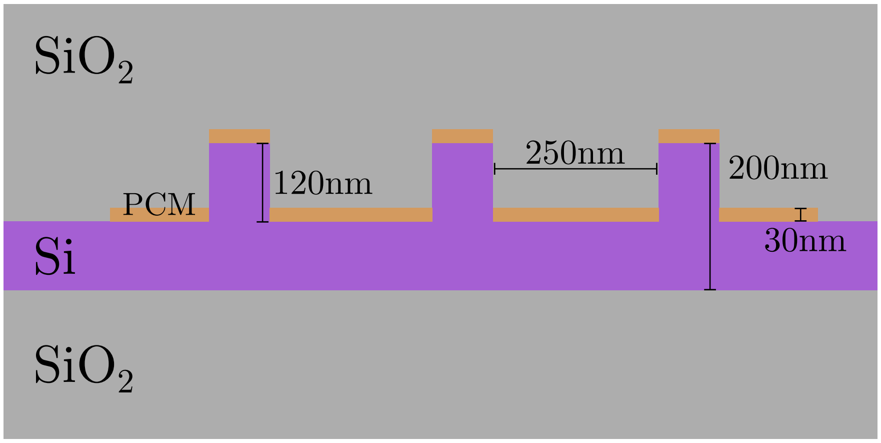

Figure S4: Cross section schematic of the 3×3 Si waveguide array inside the coupling region. Outside of this waveguides fan out to a spacing of 1µm to avoid cross-talk.

Brute force training data generation

A brute force iterative optimization is used to generate training data for this work. The algorithm is a simple approach which can be applied to any multiport device. An unperturbed device is created in the users photonic simulation software of choice, in this work Lumerical

MODE is used as a variational FDTD program. A random intensity target is selected and the starting weight is calculated as follows:

$$W = \sum_{i=1}^{n} 1 - \Delta \mathrm{T}_i \tag{1}$$

here n is the number of output ports, ΔT represents the difference in transmission between the target and current geometry. A random pixel is selected and switched between crystalline or amorphous states and the weight calculated. If the weight increases the change is saved to the database, otherwise it is reverted. While this approach is sufficient for a single port optimization, if a multi-port optimization is desired a further summation each input waveguide would be necessary.

Figure S5 shows an example single port optimization for a 0%/50%/40% splitting ratio for light injected into input channel 1. This demonstrates the minimal scattering losses, as well as the tendency of such an approach to become stuck in a local minima during the optimization. Oscillations in the transmission observed after reaching a local minima are a result of transmission results being recorded before perturbations are either accepted or rejected, after this point most pixels are rejected however the effect of their perturbation is still recorded in Figure S5.

The aforementioned data generation was run over the course of several weeks resulting in a training dataset uniformly distributed around a central point close to 0.33 (even splitting for the here studied 3 waveguide system). Figure S12 shows however that the data distribution in phase space is not so uniform. Because we are only able to apply phase delays we observe a clear peak corresponding to the phase of each matrix element in an unperturbed system. In later generations of the training data optimization, we attempted to rectify this issue by assigning random phase targets to each port and including a phase MSE term such that Equation 1 now becomes:

$$W = \mathrm{MSE}_{\phi} + \mathrm{MSE}_{A} \tag{2}$$

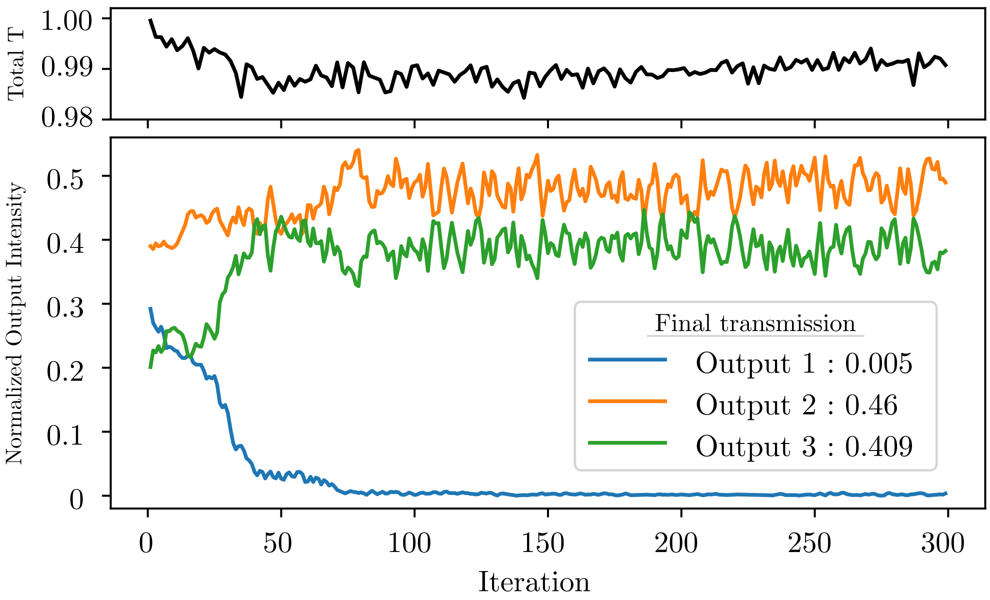

Figure S5: Example brute force optimization of a single input for a 3×3 waveguide array. The target splitting ratio (0%/50%/40%) is approached within 100 iterations before becoming stuck in a local minimum. Total device throughput, shown in the top panel, remains high as pixels are written dropping only by 0.6% across the entire optimization.

Because phase delays are always be added relative to the devices initial value, and as the optimization progresses pixels are increasingly likely to be rejected, an iterative approach is not able to create a uniform distribution of phases without disregarding a large portion of simulated data. Furthermore the addition of a phase MSE term dramatically slows the data generation rate as perturbation are more often discarded.

Training data supplementation

As discussed in the main body of this paper, we found that supplementation of the initial training dataset with a small percentage of randomly generated patterns can improve network accuracy. Figure S6 and S7 demonstrate this behaviour where network validation losses reach a minima following the addition of around 4% of randomly generated patterns. This decrease in loss corresponds to an increased average fidelity as recorded in Figure S7.

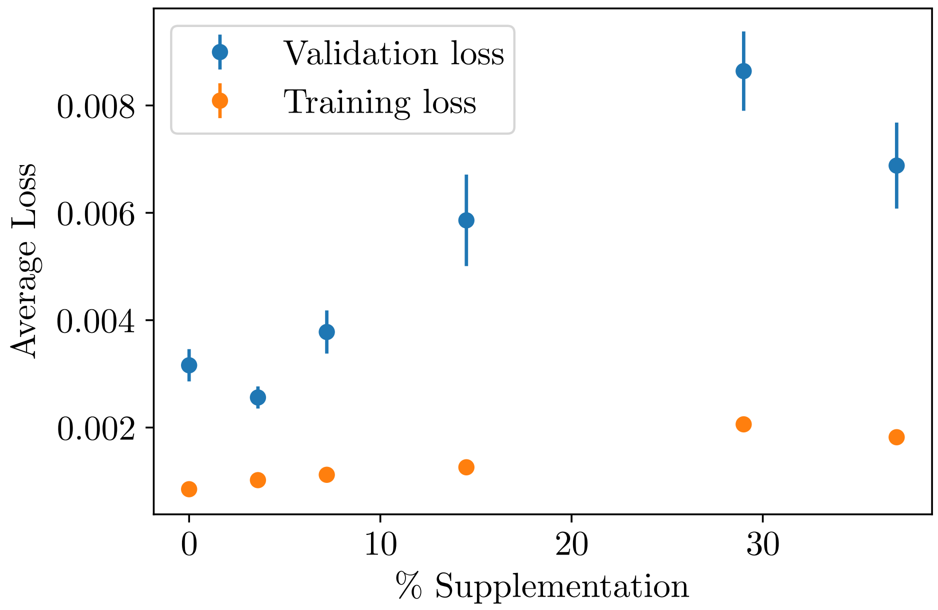

Figure S6: Effect of supplementing an amount of randomly generated ”noisy” data. Unoptimized patterns are simulated and added into the initial training data set. Here we present average training losses for the forward network, as well as losses from a separate validation dataset. Results are the average of 5 full training cycles with the standard deviation represented by the error bars.

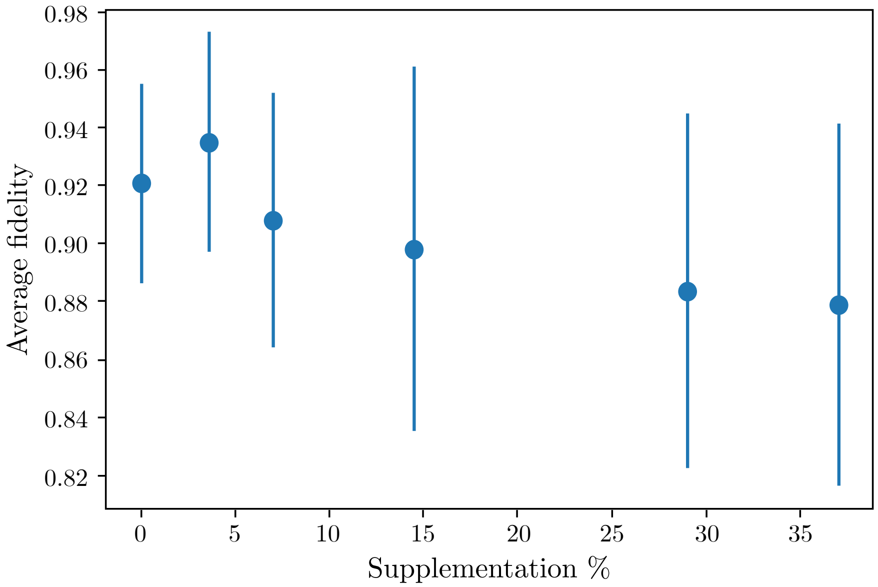

Figure S7: After dataset augmentation with varying percentages of random noisy data, each network was presented with 1000 random unitary targets for which the average fidelity is plotted above.

Amplitude Fidelity

The amplitude fidelity is used to assess the success of pixel pattern predictions which is calculated as such,

$$\mathrm{F} = \frac{1}{\mathrm{N}} \left[\mathrm{Tr}(|U^{*}| \cdot |U_{\mathrm{sim}}|)\right] \tag{3}$$

The amplitude fidelity takes into account the intensity but negates the phase agreement of the matrix under study. This is demonstrated in Figure S8 showing a strong correlation between amplitude MSE however a somewhat random distribution if we plot the same fidelities against the phase MSE.

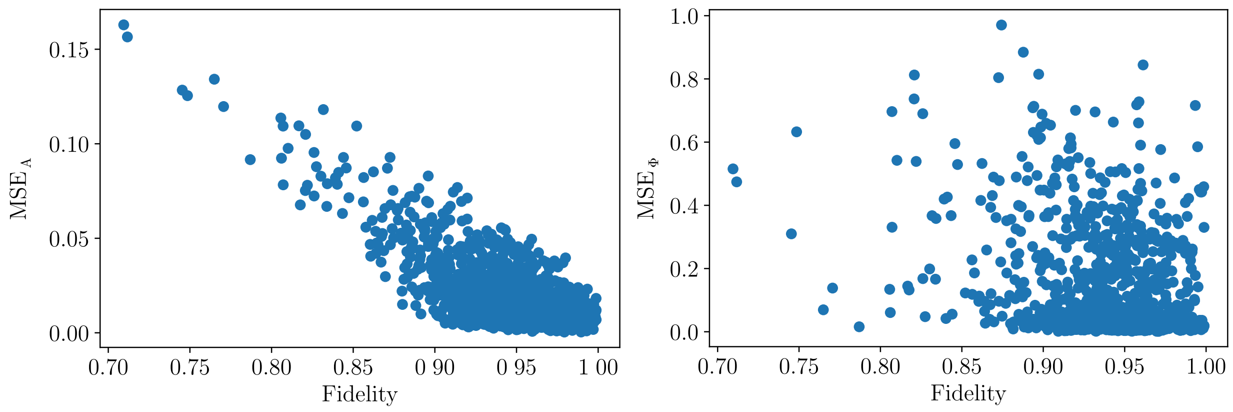

Figure S8: Correlation between amplitude fidelity and MSE for both the phase and amplitude across 1000 random matrix targets. MSE is calculated between the target and resimulation matrix with the average across all 9 elements presented here.

When attempting to improve network accuracy it is useful to consider the maximum possible performance of our approach. As the suitability of predicted patterns is assessed using the forward network, it is clear that this will define an upper limit on available performance. By investigating the fidelity between forward network predictions of the validation patterns with their "real" simulated values we can retrieve a likely estimate of the forward networks accuracy. Because this validation dataset is a sub-sample of the training data removed before training, it presents a best-case scenario where we can almost guarantee no network extrapolation is required. Figure S9 shows the recorded fidelites for such a test, with an average

value of around 0.92.

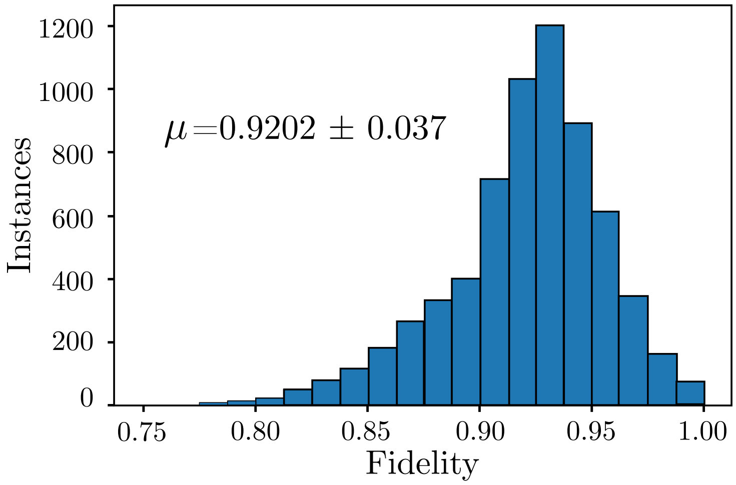

Figure S9: Amplitude fidelity distribution taken from forward network predictions for the validation dataset of pixel patterns. This prediction error is intrinsic to all presented results, and will become a limiting factor as the network is improved, outlining an upper limit on total inverse design accuracy.

Comparison with 3D simulation

In this work we use 2.5D simulations for training data generation using the variational FDTD engine within Lumerical MODE to ease the computational load and improve simulation times compared to a full 3D simulation. Using the 2.5D model simulation time of a single 3x3 transmission matrix takes around 1 minute, compared to 1 hour in the case of a full 3D simulation. While agreement is not perfect between the two regimes, this 2D approximation allows a representative case study which may then lead to further investigation of the 3D behavior which would be required to compare to a real world sample. Figure S10 shows a comparison between the 2.5D and 3D simulation of a patterned waevguide array.

Optimization stability

To ensure that network predicted patterns are suitable for experimental applications it is important to ensure optimized solutions are stable and resilient to variations in environmental

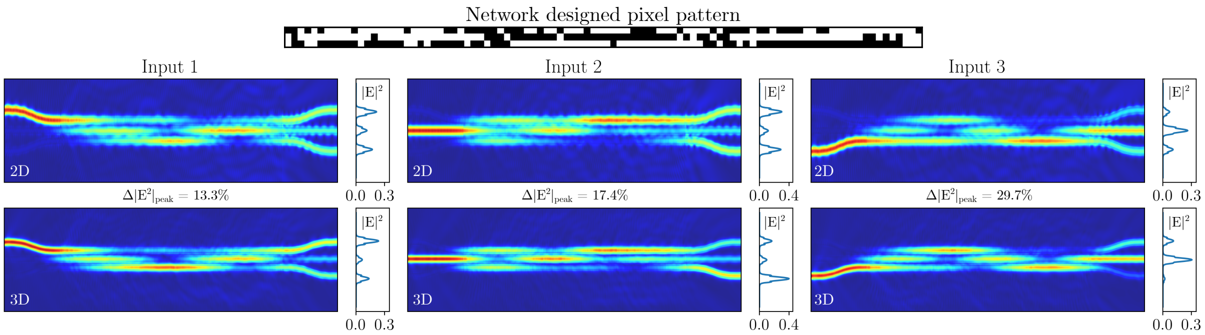

Figure S10: Comparison of the near field electric field distribution within a patterned waveguide array when using a 2.5D Variational FDTD engine as well as a full standard 3D FDTD model.

conditions. Figure S11 shows the effect on the amplitude fidelity of a simulated device to implement the 3x3 fourier matrix outlined in the main text body. We calculated the phase and amplitude variation as a result of the change in refractive index of the silicon waveguides and buried oxide layer instead. Figure S11(a) shows a small decrease in fidelity by 0.06, from 0.97 to 0.915, across the presented temperature range. Representative matrices at five different temperatures over the range from 255 K to 315 K are presented in Figure S11(c), along with the target matrix which is identical to Figure 6 in the main text. Large differences in colour are observed indicating a change in phase of the matrix elements. However, we find that a global phase shift can be factorized out of the matrix elements by taking the average of the difference of each matrix element with the same element in the target matrix. This extracted global phase, Φ_0, is plotted against temperature in Figure S11(b) and shows a linear trend from -2.5 rad to 1.8 rad over a temperature range of 80 K, corresponding to a thermo-optic global phase shift of 0.054 rad/K. Correcting each matrix by subtracting this global phase shift results in the matrices shown in Figure S11(c) with corresponding polar plots in Figure S11(d). These matrices look identical by eye and the decrease in fidelity would be mostly observed as a difference in saturation of a few percent. Overall we can see that the designed matrix has a low sensitivity to variations in temperature of several tens of K when correcting for the global phase shift.

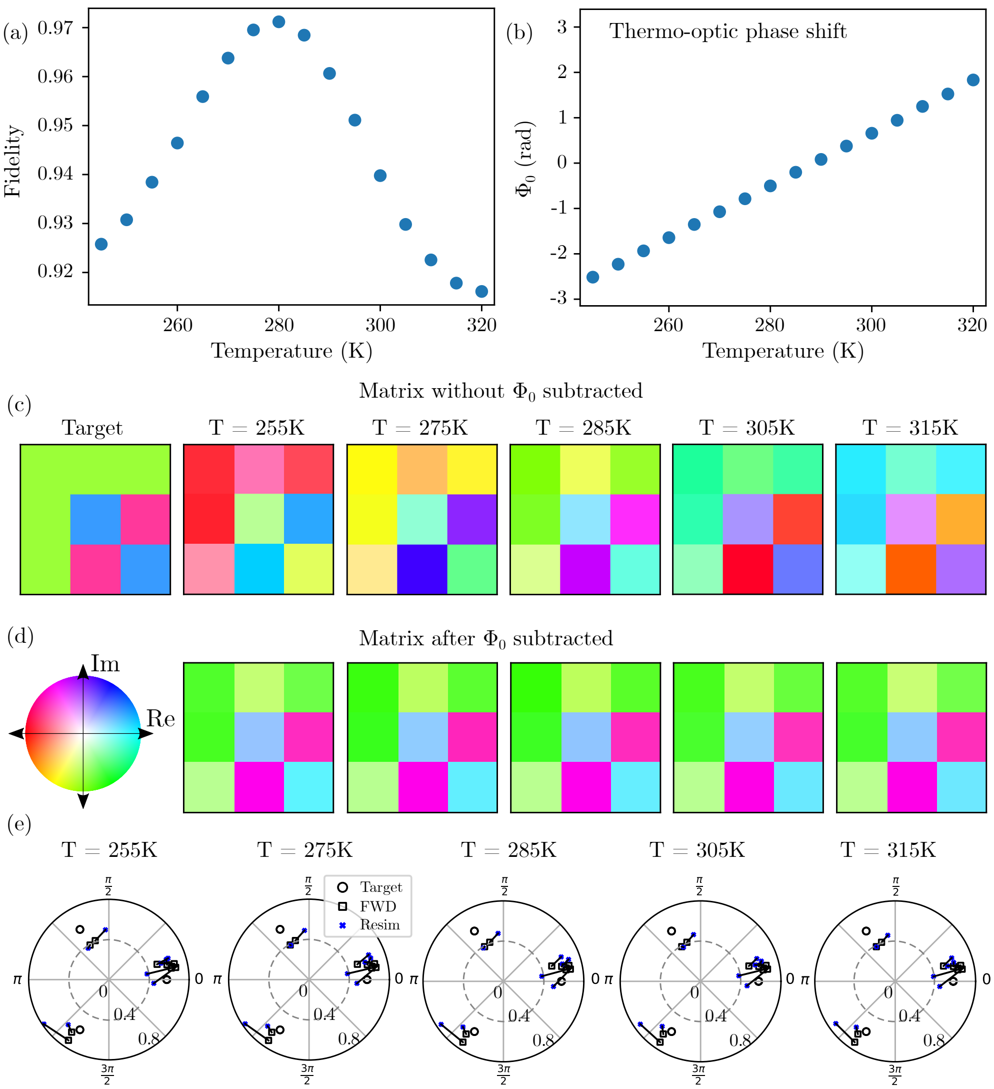

Figure S11: (a) Variation in the amplitude fidelity with simulation temperature of a patterned device designed to implement a 3×3 fourier matrix, over a temperature range from 245 K to 320 K. (b) Thermo-optic global phase shift Φ_0 extracted from the average difference in optical phase for the matrix elements. (c,d) Uncorrected transmission matrix without Φ_0 subtracted (c) and corrected by subtraction of the global phase Φ_0 (d), with corresponding polar plots (e) comparing target, forward model at room temperature and temperature-dependent simulation with global phase correction.

Training data statistics

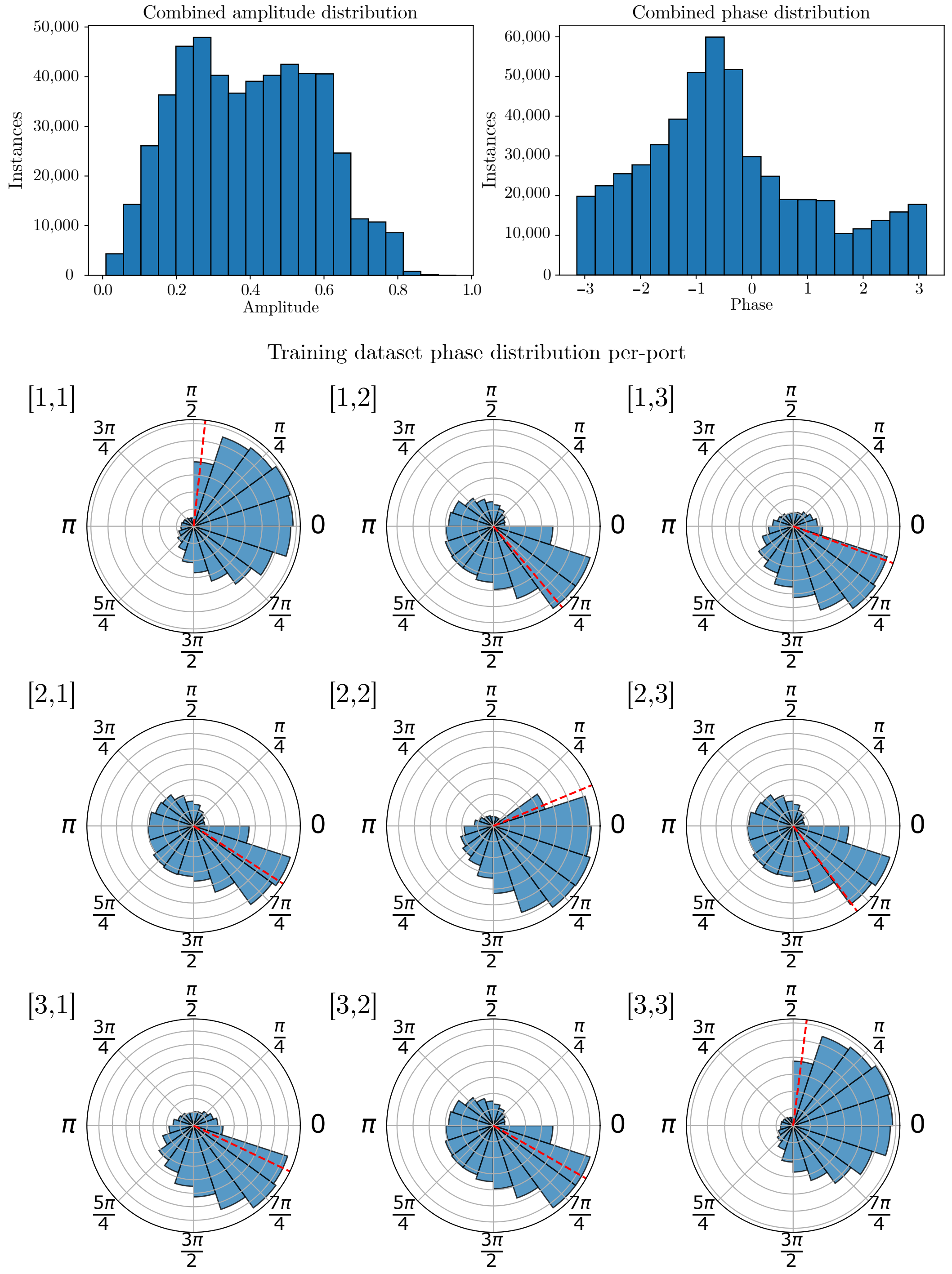

Figure S12: Phase and intensity distributions per matrix element in the training dataset. Amplitude values show a mostly normal distribution, however, as Phase values are not addressed in data generation and the training patterns are generated iteratively, the created dataset is highly biased towards the unperturbed device phase (dashed red line)

References

(1) Xie, S.; Girshick, R.; Dollár, P.; Tu, Z.; He, K. Aggregated Residual Transformations for Deep Neural Networks. 2017, 1611.05431, arXiv, (accessed 01/15/2025); https://arxiv.org/abs/1611.05431.

(2) Smith, S. L.; Kindermans, P.-J.; Ying, C.; Le, Q. V. Don't Decay the Learning Rate, Increase the Batch Size. 2018, 1711.00489, arXiv, (accessed 01/15/2025); https://arxiv.org/abs/1711.00489.

(3) Gulrajani, I.; Ahmed, F.; Arjovsky, M.; Dumoulin, V.; Courville, A. C. Improved training of wasserstein gans. Advances in neural information processing systems **2017**, 30.

(4) Kingma, D. P.; Ba, J. Adam: A Method for Stochastic Optimization. 2017, 1412.6980, arXiv, (accessed 01/15/2025); https://arxiv.org/abs/1412.6980.
